# Life engagement improvements with cariprazine in schizophrenia: a post-hoc analysis of PANSS data from short-term studies

**DOI:** 10.1093/ijnp/pyag027

**Published:** 2026-05-08

**Authors:** Réka Csehi, Péter Szabó, Zsófia Borbála Dombi, Thomas Brevig, Balázs Szatmári, Ágota Barabássy

**Affiliations:** Global Medical Division, Gedeon Richter Plc., Budapest, Hungary; Global Medical Division, Gedeon Richter Plc., Budapest, Hungary; Global Medical Division, Gedeon Richter Plc., Budapest, Hungary; Global Medical Division, Gedeon Richter Plc., Budapest, Hungary; Global Medical Division, Gedeon Richter Plc., Budapest, Hungary; Global Medical Division, Gedeon Richter Plc., Budapest, Hungary

**Keywords:** partial agonist, cariprazine, life engagement, schizophrenia

## Abstract

**Objective:**

Patient life engagement (PLE) is increasingly recognized as a meaningful treatment goal in schizophrenia, yet it remains under-researched, partly due to the lack of validated PLE measures. This post-hoc analysis evaluated the effect of cariprazine on PLE using a 14-item subset of the Positive and Negative Syndrome Scale (PANSS).

**Methods:**

Data from three 6-week, randomized, double-blind, placebo-controlled trials were pooled and analyzed post-hoc. The 14-item PANSS subset measured PLE across four domains: Cognitive, Social, Emotional, and Physical. Changes from baseline to Week 6 in PLE Total Score and domain scores were analyzed using mixed models for repeated measures. Responder rates (≥5- and ≥ 10-point reductions) were further assessed.

**Results:**

Cariprazine (n = 880; 1.5-6.0 mg/day) showed significantly greater improvements than placebo (n = 446) on PLE Total Score (LS mean difference [LSMD] = –3.23; 95% CI: −4.59 to −1.87; Cohen’s d = 0.79, *P* < .001) and across Emotional (LSMD: −0.59; 95% CI: −0.97 to −0.22; Cohen’s d = 0.49, *P* < .001), Social (LSMD: −1.13; 95% CI: −1.65 to −0.6; Cohen’s d = 0.71, *P* < .001), and Cognitive (LSMD: −1.34; 95% CI: −1.91 to −0.76; Cohen’s d = 0.77, *P* < .001) domains, and numerically greater improvement on the Physical (LSMD: −0.25; 95% CI: −0.5 to 0.01; Cohen’s d = 0.30, *P* = .071) domain. Statistically significant improvements emerged by Week 2 on the PLE Total and were sustained through Week 6. Responder analyses showed that 81% and 57% of cariprazine-treated patients met the ≥5- and ≥ 10-point thresholds, respectively, versus 64% and 43% with placebo (both *P* < .001).

**Conclusion:**

The results suggest that cariprazine is associated with significant improvements in patient life engagement in schizophrenia based on the 14-item subset of the PANSS, with early and clinically meaningful benefits across domains. These findings support the value of cariprazine in addressing patient-centered treatment goals.

**Significant outcomes**

- Cariprazine produced significantly greater overall symptom and illness-severity improvements than placebo, with large effect sizes on both PANSS Total (LSMD −7.44) and CGI-S (LSMD −0.41).
- Cariprazine showed consistently superior reductions in PLE Total and domain scores – especially Social, Cognitive, and Emotional domains – and demonstrated significantly faster and larger weekly improvements from Week 2 onward in PLE Total.
- Clinically meaningful response rates were higher with cariprazine, with 81% achieving ≥5-point improvement and 57% achieving ≥10-point improvement, both significantly outperforming placebo.

**Limitations**

- As a post-hoc analysis, it is inherently exploratory and subject to limitations of the original study designs.
- While recently validated through a robust process, this 14-item subset of the PANSS is a new tool. Its performance over longer time periods, ability to predict functional outcomes, and usefulness across diverse clinical populations still need further investigation.
- Finally, the studies could not control for external psychosocial factors that may influence life engagement, like participation in psychosocial interventions, caregiver support or follow-up with mental health professionals.

## Introduction

Meaningful recovery in schizophrenia extends beyond the mere reduction of symptoms.^1^ While symptom remission remains a crucial treatment target, there is increasing recognition that a more holistic approach should incorporate outcomes that are meaningful to patients. Individuals living with schizophrenia frequently identify improvements in autonomy, motivation, cognitive clarity, emotional and physical well-being, and social and familial relationships as priorities in their treatment journey.^1^ These perspectives have contributed to a broader shift toward recovery-oriented and patient-centered care, in which functioning, quality of life, and treatment goals that matter to patients are considered alongside symptom reduction and relapse prevention.^2^

In this context, patient-reported outcomes (PROs) have become increasingly central in psychiatric clinical trials, as they capture aspects of health and recovery that clinician-rated measures may not fully reflect.^3^ Evidence suggests that PROs complement clinician assessments and can reveal meaningful changes in daily functioning, subjective well-being, and life satisfaction that are not always captured by traditional symptom scales alone.^4^ However, PROs remain inconsistently implemented in schizophrenia trials, and many pivotal studies continue to rely primarily on clinician-rated instruments.

Within this evolving framework, patient life engagement (PLE) has emerged as a multidimensional construct encompassing emotional, social, cognitive, and physical domains of positive health and functioning.^5^ Life engagement broadly reflects vitality, motivation, reward responsiveness, cognition, and social connectedness – domains consistently identified by patients as central to recovery. Importantly, even when formal PRO instruments are not incorporated into trial designs, constructs such as life engagement – particularly when operationalized using validated composites aligned with patient priorities – may provide a meaningful approximation of patient-centered benefit within existing clinician-rated frameworks. Taken together, these developments support the inclusion of PLE in clinical research to provide a more comprehensive understanding of treatment effects from the patient’s perspective.

Despite its clinical importance, PLE remains underexplored in schizophrenia research, partly due to the lack of validated measures.^6^ Traditional psychiatric rating scales often fall short in capturing the full range of domains that comprise PLE. These scales, such as the PANSS, focus on symptom reduction but do not fully capture life engagement domains, as they were not designed for this purpose.^5^

However, there have been efforts made to derive composite measures from existing clinician-rated instruments. One example is the 11-item PANSS-based composite (PANSS-11),^7^ constructed to capture domains considered central to life engagement, including Blunted Affect (N1), Emotional Withdrawal (N2), Poor Rapport (N3), Social Withdrawal (N4), Difficulties in abstract thinking (N5), Lack of spontaneity and flow of conversation (N6), Depression (G6), and Motor retardation (G7), Disturbances of volition (G13), Preoccupation (G15) and Active social avoidance (G16). The PANSS-11 was subsequently examined in a large community sample of individuals living with schizophrenia, where life engagement scores demonstrated meaningful associations with functioning, cognitive performance, and overall illness severity.^8^ These findings provided empirical support for the construct validity of life engagement as a clinically relevant, multidimensional concept within schizophrenia research.

Building on this framework, a more recent and expanded 14-item PANSS-based subset (PANSS-14) was developed through a multi-step process that included expert consultation, interviews with individuals living with schizophrenia, and principal component analyses of clinical trial datasets.^5^ Items were selected if they were identified as relevant by at least two of these three methods, ensuring both clinical and patient-centered validity. In addition to the core negative and general psychopathology items included in the PANSS-11, the PANSS-14 incorporates additional items reflecting cognitive and thought-organization domains (eg, Stereotyped thinking [N7], conceptual disorganization [P2], and poor attention [G11]), thereby broadening the multidimensional representation of life engagement. The resulting 14-item subset demonstrated strong content validity in capturing emotional, social, cognitive, and physical domains of life engagement. Scores range from 14 (indicating best engagement) to 98 (indicating worst), with minimal clinically important differences (MCID) estimated at 5 points for small-to-moderate improvement and 10 points for large improvement.

Cariprazine is a third-generation atypical antipsychotic approved for the treatment of schizophrenia,^9^^,^^10^ depressive and manic/mixed episodes associated with bipolar I disorder^10^ and major depressive disorder as an adjunctive treatment.^10^ It exerts partial agonist activity at dopamine D2 and D3 receptors, with notably greater affinity for D3 receptors^11^ – a property thought to contribute to its efficacy in addressing negative symptoms, cognitive dysfunction, and reward-related deficits.^12^ In randomized trials, cariprazine has demonstrated superiority over comparators, including risperidone, in reducing negative symptoms,^13^ and it has shown potential in improving domains such as emotional expression, social withdrawal, and avolition – all of which are closely aligned with the theoretical framework of life engagement. In fact, a previous post-hoc analysis in patients with predominantly negative symptoms found that cariprazine significantly improved patient engagement versus risperidone, based on an 11-item subset of the PANSS.^14^

Given the importance of PLE as a treatment outcome valued by individuals with schizophrenia – and the recent development of a PANSS-derived 14-item subset to assess it – it is essential to understand how different antipsychotics affect this domain. Thus, the primary objective of this post-hoc analysis was to evaluate the effects of cariprazine, compared with placebo, on patient life engagement as measured by the 14-item PANSS subset in patients with acute exacerbation of schizophrenia. Specifically, we aimed to examine changes in PLE Total Score and domain scores over 6 weeks of treatment, the time course of improvement, and the proportion of patients achieving clinically meaningful response thresholds (≥5-point and ≥ 10-point improvement). We hypothesized that cariprazine would be associated with greater improvements in PLE compared with placebo and with earlier separation over the course of treatment. By focusing on this outcome, the analysis aims to provide insight into how cariprazine may help patients achieve treatment goals most relevant for them.

## Material and methods

### Study design and patients

In this post-hoc analysis, data were pooled from three randomized, double-blind, placebo-controlled phase II/III clinical trials (ClinicalTrials.gov identifiers: NCT00694707, NCT01104766, NCT01104779) that were conducted as part of the clinical development program of cariprazine prior to its regulatory approval for schizophrenia.^15-17^ The studies were multinational (primarily in the United States, but also in some countries of Europe, Asia, and South Africa) and multicentre (41-65 centers), conducted between 2008-2009^17^ and 2010-2011.^15^^,^^16^

All three studies followed a comparable protocol. Each trial incorporated a washout phase of up to 7 days, followed by a 6-week double-blind treatment period and a subsequent safety follow-up lasting 2–4 weeks. In all studies, participants were hospitalized during screening and the first 4 weeks of double-blind treatment; during the final 2 weeks, treatment continued either in an inpatient or outpatient setting at the investigator’s discretion.

Although the original trials evaluated cariprazine across a dose range of 1.5–9.0 mg/day, the present analysis was restricted to doses within the approved therapeutic range for schizophrenia (1.5–6.0 mg/day), specifically 1.5, 3.0, 4.5, and 6.0 mg/day.

The pooled sample consisted of adult patients experiencing an acute exacerbation of schizophrenia who met the eligibility criteria defined in the individual source trials. Key inclusion criteria comprised a Positive and Negative Syndrome Scale (PANSS) Total Score between 80 and 120 (inclusive of 120) and a Clinical Global Impressions–Severity (CGI-S) score of ≥4 (moderate severity). Major psychiatric exclusion criteria included the presence of any DSM Axis I disorder other than schizophrenia within the preceding 3 months, first-episode psychosis or schizophrenia, current alcohol or substance use disorders or positive screening tests, treatment-resistant schizophrenia, and acute suicidal risk. Treatment-related exclusions encompassed electroconvulsive therapy (ECT) within the prior 3 months (or documented non-response to ECT), recent use of depot antipsychotics (less than one dosing cycle prior to screening), clozapine treatment, and use of disallowed concomitant medications, except where explicitly permitted by protocol.

The original studies were conducted in accordance with the Declaration of Helsinki and Good Clinical Practice guidelines, and the protocols were approved by the relevant Institutional Review Boards or Independent Ethics Committees at each participating center. All participants provided written informed consent prior to enrolment.

### Assessment

The PANSS was the primary, and the Clinical Global Impressions – Severity (CGI-S) scale was the secondary outcome measure in all included studies. “PLE Total Score” was defined as the sum of the following 14 items of the PANSS^5^: Blunted affect (N1), Emotional withdrawal (N2), Poor rapport (N3), Passive social withdrawal (N4), Difficulty in abstract thinking (N5), Lack of spontaneity and flow of conversation (N6), Stereotyped thinking (N7), Conceptual disorganization (P2), Depression (G6), Motor retardation (G7), Poor attention (G11), Disturbance of volition (G13), Preoccupation (G15), and Active social avoidance (G16).

**Figure 1:**
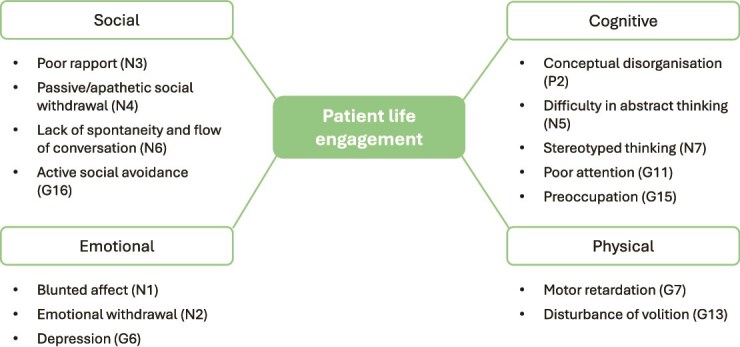
Domains of patient life engagement assessed by the PANSS 14-item subset. This conceptual figure illustrates the four core domains of patient life engagement – Emotional, Social, Cognitive, and Physical – as measured by the 14-item subset of the Positive and Negative Syndrome Scale (PANSS). Item codes indicate their original PANSS classifications (N = negative, G = general psychopathology, P = positive).

Furthermore, the 14 items were categorized into four core domains reflecting PLE: Emotional, Social, Cognitive, and Physical (Figure 1).^5^

### Statistical analysis

Descriptive statistics were used to summarize patient demographics (sex, mean age, illness duration, and age of onset). Least square (LS) mean change from baseline to Week 6 of the PANSS Total Score, CGI-S score, PLE Total Score and the four PLE Domain Scores (Social, Emotional, Cognitive, Physical) were calculated. Data were analyzed using a mixed-effects model for repeated measures (MMRM) approach with treatment, visit, and study as fixed factors, baseline as covariate, and treatment-by-visit and baseline-by-visit as interactions; an unstructured covariance matrix was used to model the covariance of within-patient scores. Between-group differences were evaluated using two-sided tests at a nominal significance level of 0.05, without adjustment for multiple comparisons. Cohen’s d effect sizes were calculated by dividing the opposite of the difference of LS mean estimates by the pooled standard deviation (residual standard deviation of the model in our case). Furthermore, weekly changes in PLE Total Scores between the cariprazine and placebo groups were analyzed, and the evaluation of the PLE response was based on two benchmarks derived from earlier MCID estimates: a decrease of ≥5 points and ≥10 points on the 14-item PANSS subset (ie, PLE Total Score).^5^ Statistical analyses were performed using R (v4.4.1; https://www.r-project.org/).

## Results

### Patient demographics and baseline characteristics

A total of 880 patients treated with cariprazine and 446 patients receiving placebo were included in the analysis (Table 1). Demographic characteristics – including age (cariprazine: 37.1; placebo: 36.9 years), sex distribution (cariprazine: 69% males; placebo: 68% males), duration of illness (11.42 vs 11.59 years), and age at disease onset (25.71 vs 25.35 years) – were similar between the two treatment groups. Baseline clinical characteristics were also well-balanced across the two treatment groups. The mean PLE Total Score at baseline was 46.57 in the cariprazine group and 46.98 in the placebo group, indicating comparable levels of PLE prior to treatment.

**Table 1:** Patient demographics and baseline clinical characteristics.

	Cariprazine (1.5-6.0 mg/d) N = 880	Placebo N = 446
**Demographics**		
Age in years, mean (SD)	37.1 (10.36)	36.9 (11.14)
Sex		
Male, n (%)	603 (69)	305 (68)
Female, n (%)	277 (31)	141 (32)
Disease duration in years, mean (SD)	11.42 (9.19)	11.59 (9.66)
Disease age of onset in years, mean (SD)	25.71 (8.41)	25.35 (9.02)
**Baseline clinical characteristics**		
PANSS Total, mean (SD)	96.52 (9.04)	96.84 (9.17)
CGI-S, mean (SD)	4.81 (0.61)	4.83 (0.64)
PLE Total, mean (SD)	46.57 (6.62)	46.98 (6.71)
PLE Domain Scores		
Emotional, mean (SD)	9.83 (2.11)	10.01 (2.12)
Physical, mean (SD)	5.57 (1.55)	5.61 (1.62)
Social, mean (SD)	13.47 (2.78)	13.87 (2.76)
Cognitive, mean (SD)	17.7 (2.9)	17.49 (3.24)

Abbreviations: SD, standard deviation; PANSS, Positive and Negative Syndrome Scale; CGI-S, Clinical Global Impressions – Severity; PLE, patient life engagement (measured by the 14-item PANSS subset).

### Efficacy outcomes

#### Efficacy outcomes at Week 6

The cariprazine group showed significantly greater overall symptom [PANSS Total: LS mean difference (LSMD): −7.44 (95% CI: −10.24 to −4.64; Cohen’s d = 0.95, *P* < .001] and severity [CGI-S: LSMD: −0.41 (95% CI: −0.58 to −0.24; Cohen’s d = 0.81, *P* < .001)] improvement than the placebo group.

**Figure 2:**
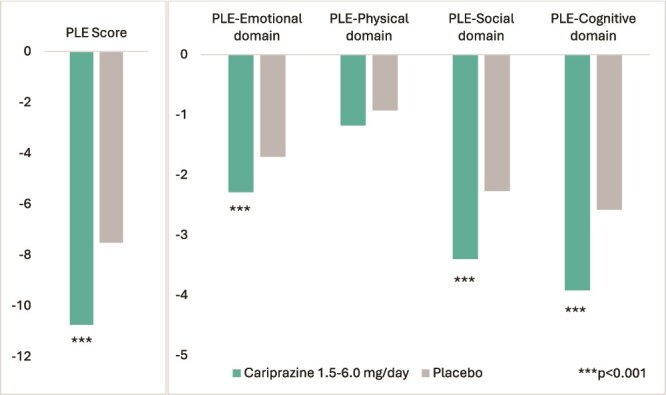
Least square (LS) mean change from baseline in PLE Total and Domain Scores after 6 weeks of treatment with cariprazine vs. placebo.

For the Total PLE Score, the cariprazine group demonstrated significantly greater improvement (LS mean change: −10.75; SE: 0.3) compared to placebo (LS mean change: −7.52; SE: 0.39), yielding a LSMD of −3.23 (95% CI: −4.59 to −1.87; Cohen’s d = 0.79, *P* < .001) (Figure 2). Across PLE domains, statistically significant group differences were observed for the Emotional (LSMD: −0.59; 95% CI: −0.97 to −0.22; Cohen’s d = 0.49, *P* < .001), Social (LSMD: −1.13; 95% CI: −1.65 to −0.6; Cohen’s d = 0.71, *P* < .001), and Cognitive domains (LSMD: −1.34; 95% CI: −1.91 to −0.76; Cohen’s d = 0.77, *P* < .001) (Figure 2). The cariprazine group had a numerically greater improvement on the Physical domain (LSMD: −0.25; 95% CI: −0.5 to 0.01; Cohen’s d = 0.30, *P* = .071) (Figure 2).

#### Weekly changes in PLE total score

**Figure 3:**
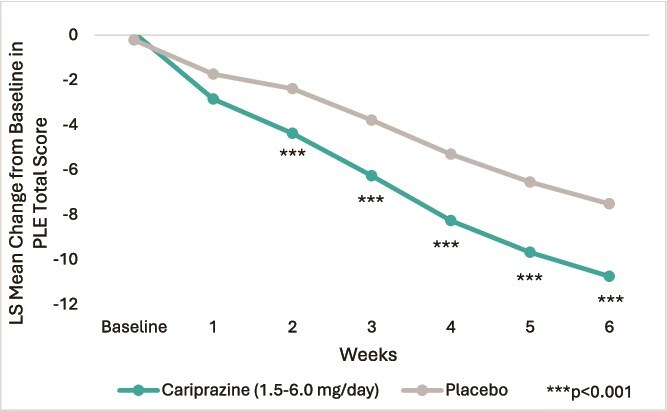
Least square (LS) mean change from baseline in patient life engagement (PLE) Total score by week. The figure illustrates the weekly LS mean change from baseline in PLE Total score over a 6-week treatment period for the cariprazine (n = 880) and placebo (n = 446) groups.

Looking at the weekly LS mean changes from baseline in PLE Total Scores (Figure 3), patients receiving cariprazine exhibited a more pronounced and rapid reduction at every time point compared to those receiving placebo. Cariprazine demonstrated a clear and statistically significant advantage over placebo from Week 2 onwards (all *P* < .001).

#### Responder rates

**Figure 4:**
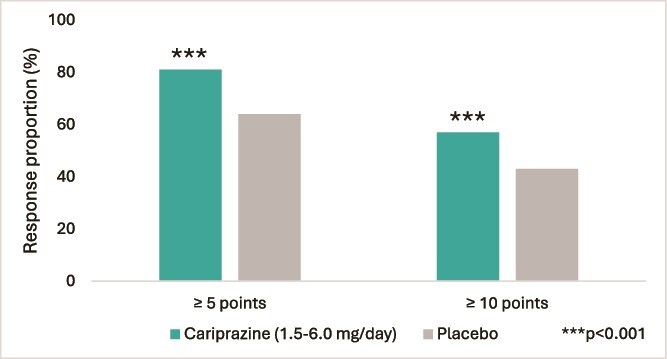
Proportion of responders achieving ≥5-point and ≥ 10-point improvement. The figure illustrates the proportion of patients who achieved clinically meaningful improvements of ≥5 points and ≥ 10 points on the patient life engagement Total score, comparing the cariprazine (1.5–6.0 mg/day) and placebo groups.

Among patients who achieved clinically meaningful improvements of ≥5 points, 81% of patients treated with cariprazine met the response criterion, significantly exceeding the 64% observed in the placebo group (*P* < .001) (Figure 4). Similarly, at the ≥10-point threshold, 57% of cariprazine-treated patients showed improvement in PLE, versus 43% with placebo (*P* < .001) (Figure 4).

## Discussion

This post-hoc analysis of pooled data from three short-term randomized controlled trials provides new insights into the effects of cariprazine on patient life engagement, a construct increasingly recognized as vital in evaluating recovery in schizophrenia.

The robust effect of cariprazine on the PANSS 14-item PLE Total Score [effect size (ES): Cohen’s d = 0.79] indicates a meaningful therapeutic benefit. In fact, changes in PLE Total Score became evident early in treatment, with significant separation from placebo observed as early as Week 2. This early response may have important implications for clinical decision-making, especially considering that early improvement is often predictive of longer-term outcomes in schizophrenia treatment.^18^ Furthermore, it is notable that the cariprazine treatment group showed greater improvements across three PLE domains compared to placebo, with particularly strong effects in the Cognitive (ES = 0.77), Social (ES = 0.71), and Emotional (ES = 0.49) domains. These findings align with previous findings on cariprazine’s effectiveness in related aspects of cognition^19^^,^^20^ and negative symptoms^13^^,^^21^^,^^22^ as well as cariprazine’s known pharmacodynamic profile^11^^,^^23^ – particularly its D3-preferring partial agonist activity – which is hypothesized to target cognitive and motivational deficits.^24^ Additionally, in a review by Morozov and colleagues,^25^ cariprazine was termed as a socializing drug, emphasizing its ability to improve the negative, affective and cognitive symptom profile of patients and therefore enabling them to be more social.^25^ Additionally, this aspect is recurring in case reports, where patients are described to be more open to their surroundings.^26^^,^^27^

The significantly higher responder rates observed in the cariprazine group, compared to placebo, further support the clinical relevance of these findings. Achieving a ≥ 5- or ≥ 10-point improvement on the PLE Total Score reflects a level of symptom reduction that is likely to be meaningful for both patients and clinicians.^5^ The fact that 81% of patients treated with cariprazine reached the ≥5-point threshold, and 57% achieved a ≥ 10-point improvement, underscores the efficacy of cariprazine in this population. These results suggest that cariprazine may not only produce statistically significant changes but also deliver clinically relevant benefits that may translate into improved daily functioning and quality of life for patients. Furthermore, the clear separation from placebo across both responder thresholds highlights the reliability and consistency of cariprazine’s therapeutic effects.

These findings are consistent with previous research on cariprazine and life engagement. A previous post-hoc analysis^14^ investigated the effectiveness of cariprazine on PLE in a different population – patients with predominantly negative symptoms of schizophrenia. In that study, life engagement was assessed using 11 items from the PANSS (PANSS-11) over 26 weeks of treatment with either cariprazine or risperidone. Cariprazine demonstrated significantly greater improvement in PANSS-11 (N1, N2, N3, N4, N5, N6, G6, G7, G13, G15, G16) Total Score compared to risperidone (LSMD: –1.79; *P* = .004), with most individual items showing statistically or numerically greater effects. These findings suggest that cariprazine may enhance life engagement – not only in patients with acute schizophrenia, as shown in the current analysis, but also in those with predominant negative symptoms, and importantly, in comparison to another antipsychotic medication frequently used in psychiatric clinical practice.

There have been some studies exploring the impact of antipsychotics on PLE. For instance, a post-hoc analysis of the JEWEL study assessed the effects of lurasidone using the PANSS-11 Life Engagement subscale and found that lurasidone significantly outperformed placebo (LSMD = –1.6; *P* = .006; effect size = 0.27) in patients with schizophrenia.^28^ Similarly, a post-hoc analysis by Ismail and colleagues^29^ examined brexpiprazole and reported superior outcomes compared to placebo (*P* < .05; standardized effect size = –0.31) in an acute schizophrenia population, also using the PANSS-11 as a measure of PLE.

Furthermore, to date, only one comparable post-hoc study – with brexpiprazole^30^ – has used the 14-item PANSS subset to assess patient life engagement (PLE). In that study – similarly to the present analysis – brexpiprazole doses were pooled, and only patients who received the FDA-approved dose range of 2.0–4.0 mg/day were included in the analysis. Results showed that brexpiprazole outperformed placebo in both the short- and long-term: brexpiprazole achieved a LSMD in PLE Total Score of –2.58 (Cohen’s d = 0.28), compared to –3.23 for cariprazine (Cohen’s d = 0.79) in the present analysis, indicating a larger effect size for cariprazine. However, cross-study comparisons should be interpreted with caution due to differences in study populations and designs – direct comparisons would be needed to confirm this.

An important aspect to highlight is that since the PLE-14 is derived from selected PANSS items, it inherently overlaps with symptom dimensions, particularly negative and general psychopathology domains. Therefore, improvements in PLE scores are not entirely independent of overall symptom change. However, the rationale for this composite is not to replace traditional symptom measures but to reorganize selected items into a multidimensional construct aligned with domains patients identify as meaningful to recovery, including motivation, social connectedness, and cognitive clarity. In this way, the PLE framework offers a patient-centered lens through which symptom change may be interpreted, while acknowledging its basis within a clinician-rated scale.

It is important to note, however, that this study is not without limitations. As a post-hoc analysis, it is inherently exploratory and subject to limitations of the original study designs.^31^ Furthermore, in this analysis, all cariprazine dosing arms (1.5–6.0 mg/day) were pooled into a single group, which may obscure important dose–response relationships or the identification of optimal therapeutic doses for improving PLE. Another limitation relates to the assessment method. Although the 14-item PANSS-based PLE composite was developed to reflect domains meaningful to patients, it remains a clinician-rated instrument derived from a symptom scale rather than a direct patient-reported outcome measure. Consequently, it may not fully capture patients’ subjective experiences of engagement, motivation, and well-being. Future research would benefit from incorporating both clinician-rated and patient-reported measures to provide a more comprehensive and person-centred evaluation of life engagement. Furthermore, while recently validated through a robust process, this 14-item subset of the PANSS is a new tool. Its performance over longer time periods, ability to predict functional outcomes, and usefulness across diverse clinical populations still need further investigation. Replicating these findings in future studies will be important to confirm the scale’s reliability and broader clinical relevance. Finally, the studies could not control for external psychosocial factors that may influence life engagement, like participation in psychosocial interventions, caregiver support or follow-up with mental health professionals. Future studies should consider these contextual variables to better understand the multifaceted nature of PLE improvement. Taken together, while the results are encouraging and support the utility of cariprazine in enhancing patient life engagement, these limitations underscore the need for cautious interpretation and highlight directions for future research.

To conclude, this post hoc analysis suggests that cariprazine might be associated with meaningful improvements in patient life engagement – an outcome that captures what many individuals with schizophrenia value most. By showing both early and sustained benefits across various PLE domains, like emotional, social, and cognitive functioning, cariprazine represents a promising pharmacologic option for clinicians helping patients to achieve relevant goals with their treatment. Importantly, while the present analysis demonstrates clear benefits of cariprazine, future studies should evaluate whether improvements in PLE predict longer-term functional outcomes, such as employment and independent living. Further research might also explore whether certain patient subgroups (eg, those with predominant negative symptoms or early phase illness) derive particular benefit in life engagement from their treatment.

## Data Availability

The datasets analyzed in this study originate from company-sponsored clinical trials and are not publicly available. Qualified researchers may submit data access requests to the sponsor in accordance with applicable policies via emailing the corresponding author.
